# Inductive Power Transfer Coil Misalignment Perception and Correction for Wirelessly Recharging Underground Sensors

**DOI:** 10.3390/s25020309

**Published:** 2025-01-07

**Authors:** John Sanchez, Juan Arteaga, Cody Zesiger, Paul Mitcheson, Darrin Young, Shad Roundy

**Affiliations:** 1 Department of Mechanical Engineering, University of Utah, Salt Lake City, UT 84112, USA; 2 Department of Electrical and Electronic Engineering, Imperial College London, London SW7 2AZ, UK; 3 College of Agriculture and Applied Sciences, Utah State University, Logan, UT 84322, USA; 4 Department of Electrical and Computer Engineering, University of Utah, Salt Lake City, UT 84112, USA

**Keywords:** agricultural soil sensing, inductive power transfer, Monte Carlo methods, machine learning, power transfer coil misalignment, wireless power transfer, wireless underground sensor networks

## Abstract

Field implementations of fully underground sensor networks face many practical challenges that have limited their overall adoption. Power management is a commonly cited issue, as operators are required to either repeatedly excavate batteries for recharging or develop complex underground power infrastructures. Prior works have proposed wireless inductive power transfer (IPT) as a potential solution to these power management issues, but misalignment is a persistent issue in IPT systems, particularly in applications involving moving vehicles or obscured (e.g., underground) coils. This paper presents an automated methodology to sense misalignments and align IPT coils using robotic actuators and sequential Monte Carlo methods. The misalignment of a Class EF inverter-driven IPT system was modeled by tracking changes as its coils move apart laterally and distally. These models were integrated with particle filters to estimate the location of a hidden coil in 3D, given a sequence of sensor measurements. During laboratory tests on a Cartesian robot, these algorithms aligned the IPT system within 1 cm (0.025 coil diameters) of peak lateral alignment. On average, the alignment algorithms required less than four sensor measurements for localization. After laboratory testing, this approach was implemented with an agricultural sensor platform at the Utah Agricultural Experiment Station in Kaysville, Utah. In this implementation, a buried sensor platform was successfully charged using an aboveground, vehicle-mounted transmitter. Overall, this work contributes to the field of underground sensor networks by successfully integrating a self-aligning wireless power delivery system with existing agricultural infrastructure. Furthermore, the alignment strategy presented in this work accomplishes coil misalignment correction without the need for complex sensor or coil architectures.

## 1. Introduction

**Figure 1:**
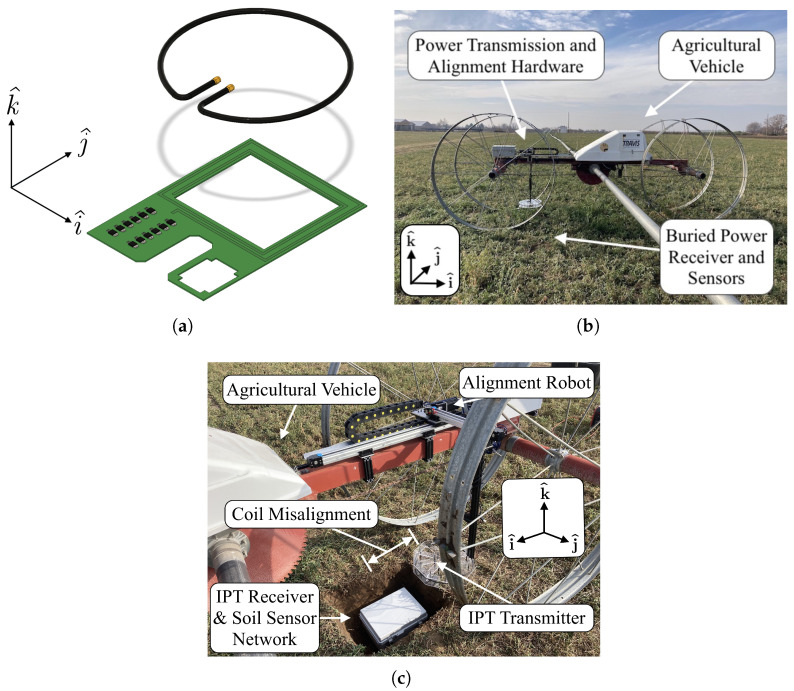
Inductive power transfer coil misalignment: (**a**) misalignment between IPT coils used for recharging underground sensors, (**b**) field implementation of the alignment hardware developed in this work, and (**c**) misalignment in field implementations of IPT. In the depicted field implementation, the IPT system and misalignment correction hardware are affixed to an agricultural wheel line mover capable of moving forward and reverse in the $\hat{i}$ direction. Lateral misalignment occurs in the $\hat{i}$ and $\hat{j}$ directions of (**a**). Distal separation/misalignment of the coils occurs in the $\hat{k}$ direction.

Persistent droughts, changes in water use policy, and efforts toward resource conservation are factors that impact water accessibility in the agricultural sector. Precision agricultural sensor networks have repeatedly proven effective at reducing water waste without sacrificing crop productivity [1]; however, farmers’ adoption of such sensor technologies has often proven slow due, in part, to concerns about the practicality of these sensor installations [2]. Sensor network obtrusiveness, complexity, and ease of use are often among the most cited concerns keeping farmers from incorporating such technology in their fields [3]. These concerns are not unwarranted, given that these sensor networks are frequently supported by extensive aboveground hardware that is easily damaged or stolen and often interferes with routine field operations. Many of these issues can be resolved by burying the entire installation underground as with increasingly popular wireless underground sensor networks (WUSNs) [4,5,6]. However, this paradigm introduces additional challenges, with power management being one of the most cited issues. While WUSNs and other embedded agricultural technologies are often built around low-power hardware, they are still frequently powered using batteries [5]. As such, battery selection becomes an issue in balancing energy capacity, survivability, and economic costs. Regardless of battery selection, almost any practically specified battery will require recharging. Recharging currently requires farmers to find (i.e., localize) and dig up the buried equipment. Both processes are time-consuming and require strenuous physical labor. Prior works have suggested vehicle-driven wireless recharging as an effective solution to this issue of power management, given innovations in the field of wireless power transfer (WPT) have provided a new framework for transferring power to inaccessible sensor networks [7,8,9,10,11]. Attempts at recharging such systems have contributed to the development and implementation of many successful WPT strategies (radio-based [12], microwave-based strategies [13], inductive-based strategies [14], etc.). Inductive power transfer (IPT) solutions (Figure 1) have proven especially promising due to their capacity to transfer large amounts of power [15], operate at comparably high efficiencies [16], and do so without significant safety risks to operators [17]. However, regardless of the WPT strategy, inaccessibility often leads to the inevitable problem of misalignment between the transmitter (often called “primary” in the context of IPT) and receiver (the “secondary”) ends of the wireless power link. Misalignment (see Figure 1a) negatively influences IPT systems because it reduces the coils’ mutual inductance $L_{ps}$ and reduces power transmission effectiveness. Accordingly, coil misalignment is often one of the limiting factors in applications of IPT [18,19]. Since many mobile IPT applications (Figure 1b) often rely on batteries as the power source for the transmitter, any reduction in efficiency reduces the amount of transferable power. Similarly, the diminution of power transfer magnitude will increase recharge mission duration, which may not be permissible under a given set of vehicle operating conditions.

Most works examining misalignment compensation have focused on dynamically tuning the primary-side hardware, which is typically a type of resonant power converter, e.g., Class E or full-bridge inverters [20,21,22]. While control methods differ based on hardware topology, many focus on controlling the switching frequency or the pulse width of the inverter’s output signal [22]. However, transmitter tuning realistically only works for minor alignment errors relative to the diameter of the coils and compensates for misalignment rather than correcting, i.e., attempts to maximize efficiency under the misaligned coupling conditions [18]. An approach focusing instead on misalignment correction is position control of the IPT system’s coils. In this approach, a sensor system detects position errors, and a robot or vehicle physically aligns the IPT coils. Unfortunately, the symmetry of the magnetic field about the transmitter means a single estimate of misalignment based on inductive coupling cannot easily be broken into $\hat{i}$, $\hat{j}$, and $\hat{k}$ contributions. This fact leads to an issue of state observability and limits the viability of traditional feedback controllers. Prior research has mainly proposed two potential solutions to this issue: optimization and multi-coil arrays [23,24]. While numerical optimization techniques have proven capable of physical alignment [8,25], the issue of state observability still negatively impacts their performance when applied to two-coil IPT systems (one transmitter, one receiver). Because misalignment measurements cannot be decomposed into directional contributions, these solutions often rely on approaches called “derivative-free” methods, such as coordinate descent, that can take many iterations to approach a solution and are not necessarily guaranteed to converge. The multi-coil array approaches, such as multiple-input–multiple-output (MIMO) or multiple-input–single-output (MISO) IPT [13], seek to eliminate the issue of state observability by adding multiple “sense” coils to the power transfer link. Each of these coils acts like an individual IPT receiver, and with an array of them, coupling-based misalignment estimates can be broken down into $\hat{i}$, $\hat{j}$, and $\hat{k}$ components. Some multi-coil solutions have even been paired with resonant tuning hardware for additional gains in efficiency [26]. Unfortunately, these muti-coil solutions do not scale well with larger coil diameters (added size and weight), require additional complex hardware, and have an increased parasitic power draw on the IPT link (reduced efficiency). Another limitation of this approach is that it requires additional data telemetry communication if the transmitter end of the IPT link is mobile instead of the receiver.

This paper presents a novel methodology for correcting coil misalignment in inductive power transfer systems for the purpose of recharging inaccessible sensor networks. To address the abovementioned issues in existing solutions, this alignment methodology must detect and physically correct for misalignments, work on both sides of an IPT transmission link, i.e., correct for misalignments using either transmitter or receiver data exclusively, and accomplish these tasks without complex coil topologies. Section 2 of this paper focuses on perceiving and correcting IPT coil misalignment using a single IPT coil pair. Perceived misalignments are corrected using sequential Monte Carlo particle filters and a path planning routine. This statistical approach addresses the state observability issue common in current misalignment correction approaches. The effectiveness and speed of this alignment approach are benchmarked extensively in Section 3 of this work. The results of these tests and a field test demonstrating effective power transfer through the soil to an underground agricultural soil sensing platform are discussed in Section 4. In this field implementation, the wireless power transfer system and alignment correction hardware are affixed to an agricultural wheel line mover. This paper concludes Section 5 by remarking on the overall viability of this methodology. Overall, the work presented in this paper contributes to the field of underground sensor installations by addressing one of the largest barriers to effective wireless recharging, i.e., power transfer coil misalignment.

## 2. Materials and Methods

Under the agricultural operational scenario discussed in this work, it is quite plausible that the underground sensor system may need to operate for several weeks or months without recharging due to variances in crop management and scheduling. The high-power transmission capabilities of the IPT system developed in this work facilitate rapid, high-energy recharging of the coupled underground hardware for additional convenience to equipment operators and as a protective measure against infrequent charge missions. The following subsections detail the design of the IPT system, robotic alignment hardware, and the coil misalignment correction algorithm.

### 2.1. Inductive Power Transmission Hardware and Perception of Coil Misalignment

IPT applications use various transmission hardware architectures and form factors to achieve wireless power transmission. System architectures are typically selected based on multiple factors, but operating frequency, desired power output, and size requirements are the most common factors influencing system design. In the MHz regime, most systems rely on resonant power converters to generate the oscillating magnetic field required for IPT. Because IPT systems vary so much in hardware design, methods of perceiving misalignment may need to differ slightly based on the specifics of the hardware.

**Figure 2:**
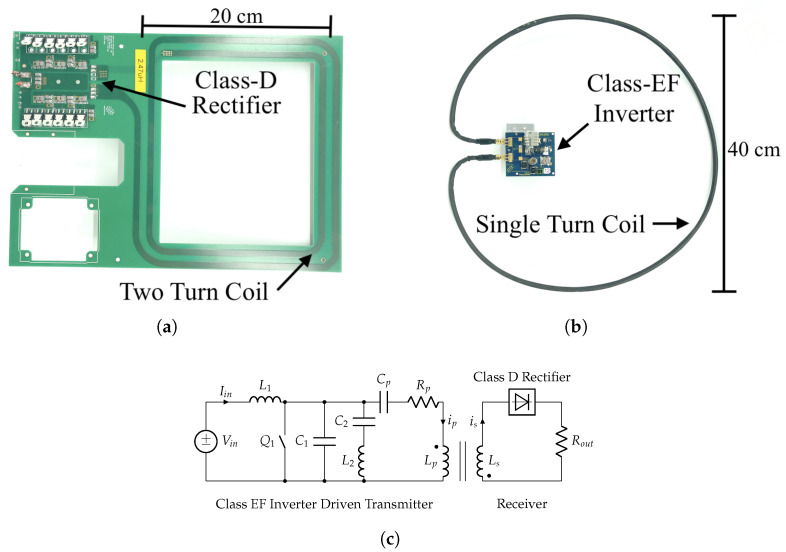
Inductive power transfer hardware: (**a**) receiver, (**b**) transmitter, and (**c**) simplified circuit of a Class EF-driven inductive power transfer system. This inverter is driven using the DC voltage input $V_{in}$.

The IPT transmitter used in this work (Figure 2b) is based on a 13.56 MHz load-independent Class EF inverter [16], a type of single-switch resonator. This inverter excites a 40 cm diameter copper-pipe coil to generate a time-varying magnetic field. The coupled receiver seen in Figure 2a uses a Class D voltage multiplier rectifier and 20 cm width PCB coil (square; 2 turns) to collect power from the transmitter. While typically used to charge batteries or supercapacitor banks, the receiver in this work is terminated with a 200 Ohm rheostat to reduce the number of influencing factors on test data. The shapes of the coils were selected for the field application presented in [14]. Given the loading conditions and DC excitation voltage used in this work (40 VDC; labeled $V_{in}$ in Figure 2c), the IPT system can transfer 10 Watts of power at 40% efficiency at perfect concentric alignment [27] and 20 cm of distal separation (distal separation is based on application requirements). Under different operating conditions, power transfer magnitude and efficiency can be improved (greater than 40 W and 80% efficiency [14]) by increasing the coupling factor, increasing the DC excitation voltage, and using a power-hungry load.

#### 2.1.1. Misalignment Estimation with a Class EF Inverter

Coil misalignment negatively impacts IPT mainly because of its effect on the system’s magnetic coupling factor *k*. As the coils of an IPT system move apart laterally, the mutual magnetic flux between the coils drops significantly. Without any additional changes to the system, Faraday’s law states that the induced EMF in the receiver will decrease in proportion to the change in flux. While this effect negatively impacts IPT system performance, it provides a useful means of misalignment estimation [28]. However, many applications of IPT using mobile robotics focus on mobile transmission hardware that charges stationary receivers [29]. Thus, the induced voltage on the receiver must be measured via the transmitter alone, which is challenging and heavily architecture-dependent. Because the IPT hardware used in this work is based on a Class EF inverter, methods of characterizing misalignment via changes in induced voltage will focus on this specific topology.

Measuring induced voltage on a Class EF-driven IPT system has been discussed in [30]. Figure 2c shows a simplified circuit schematic of a load-independent Class EF inverter and the corresponding receiver circuitry. The transmitter coil is represented by an equivalent capacitance $C_{p}$, resistance $R_{p}$, and inductance $L_{p}$. Inductor $L_{p}$ is magnetically coupled to the receiver coil $L_{s}$ with the coupling coefficient of *k*. On the receiver, voltage probes can be attached parallel to the load $R_{out}$ to measure the induced voltage $V_{Rout}$ directly. Estimating the induced voltage with the transmitter alone is more challenging. Unlike other topologies (namely Class E and Φ inverters), load-independent Class EF inverters produce a constant magnitude current $i_{p}$ in the primary coil instead of a constant voltage [16]. Because Class EF inverters are fed a constant input voltage source $V_{in}$, the DC current $I_{in}$ changes based on shifts in the load reflected from the receiver circuitry. If $R_{out}$ is held constant, as in this work, changes in magnetic coupling *k*, i.e., misalignment, will have the largest impact on $I_{in}$. Based on these relationships, the induced voltage in the secondary coil and, therefore, misalignment can directly be estimated as a function of the inverter’s DC current $I_{in}$ if the receiver hardware is inaccessible or does not support rapid data telemetry.

#### 2.1.2. Numerically Modeling Misalignment with a Class EF Inverter

In this work, numerically modeling the IPT system helps predict the behavior of the signals $I_{in}$ and $V_{Rout}$ under various conditions. The value of predicting these signals is related to two critical tasks in the coil alignment process: hardware design and control signal prediction. As the overall power delivery system is intended for embedded field applications, data-logging equipment must be purpose-built and cannot rely on benchtop equipment such as oscilloscopes and voltage supplies. The signals output by this numerical model influence design factors such as analog-to-digital converter selection, filter design, and power requirements. While numerous environmental factors, e.g., coupling with metallic structures, can impact these models’ accuracy, the IPT system’s misalignment response should follow the same general trends as the model. If sufficiently accurate, control signals can be drawn directly from the model and implemented on the alignment hardware, though the viability of such a scenario requires further validation. The control signals $I_{in}$ and $V_{Rout}$ can be estimated by beginning with the Biot–Savart law:

(1) $$B\left(r\right)=\frac{\mu_{r}}{4\;\pi }\int_{C}\frac{i_{p}\;l\times r^{\prime }}{{r^{\prime }}^{3}}$$

where r represents a Cartesian coordinate in space, B(r) the magnetic field vector at point r, $\mu_{r}$ the transmission media’s relative magnetic permeability, $i_{p}$ the current through the transmitter coil, vector dl a differential section of the transmitter coil, l the coordinate of dl, and $r^{\prime }=r-l$. The relative magnetic permeability of air, water, soil, rocks, and minerals is effectively identical ($u_{r}\approx 1$ [31]) and, therefore, can be held constant for the purposes of this work. The Biot–Savart law can be applied to a three-dimensional grid of coordinates in a computing environment such as MatLab to form the basis of this numerical model. The goal of this initial step is to estimate the shape and magnitude of the magnetic field that forms around the IPT transmitter. With a grid of precalculated B vectors, the misalignment and inclination angle of the receiver coil can be easily adjusted without significant reformulation. Once B is calculated for a sufficient number of points in space, the magnetic flux through the receiver $\Phi_{b}$ can be calculated using:

(2) $$\Phi_{b}\left(t\right)={\;\int \int \;}_{\Sigma }B\left(t\right)\cdot dA=B_{avg}\;\sin\left(\omega \;t\right)\cdot A$$

where A is the area vector of the receiver about the closed path Σ, and $B_{avg}$ is the average magnetic field strength through Σ. In an inductive power transfer system, B(t) is created by the transmitter by oscillating its current $i_{p}$ at circular frequency ω. Once magnetic flux is calculated, the voltage induced in the receiver $U_{s}$ can be calculated using Faraday’s law:

(3) $$U_{s}=-N\;\frac{d\Phi_{b}}{dt}=-\omega \;N\;B_{avg}\;\cos\left(\omega \;t\right)\cdot A$$

with *N* representing the number of coil turns in the receiver. Mutual inductance $L_{ps}$ and the coupling factor *k* can optionally be calculated using Equations (4) and (5). In these equations, $L_{p}$ and $L_{s}$ are the self-inductances of the transmitter and receiver coils, respectively.

(4) $$L_{ps}=\frac{U_{s}}{j\;\omega \;i_{p}}$$

(5) $$k=\frac{L_{ps}}{\sqrt{L_{p}\;L_{s}}}$$

Once $U_{s}$ is obtained, the power output dissipated by the receiver’s load $P_{out}$ can be calculated using:

(6) $$P_{out,\;rms}=\frac{V_{Rout}^{2}}{R_{out}}\approx \frac{U_{s,rms}^{2}}{R_{out}}$$

where $R_{out}$ is the resistance of the load, and $V_{Rout}$ is the DC voltage output by the receiver’s rectifier. Finally, $I_{in}$ can be calculated using the following relationship where η being the power transmission efficiency of the inverter.

(7) $$I_{in}=\frac{P_{out}}{\eta \;V_{in}}$$

**Figure 3:**
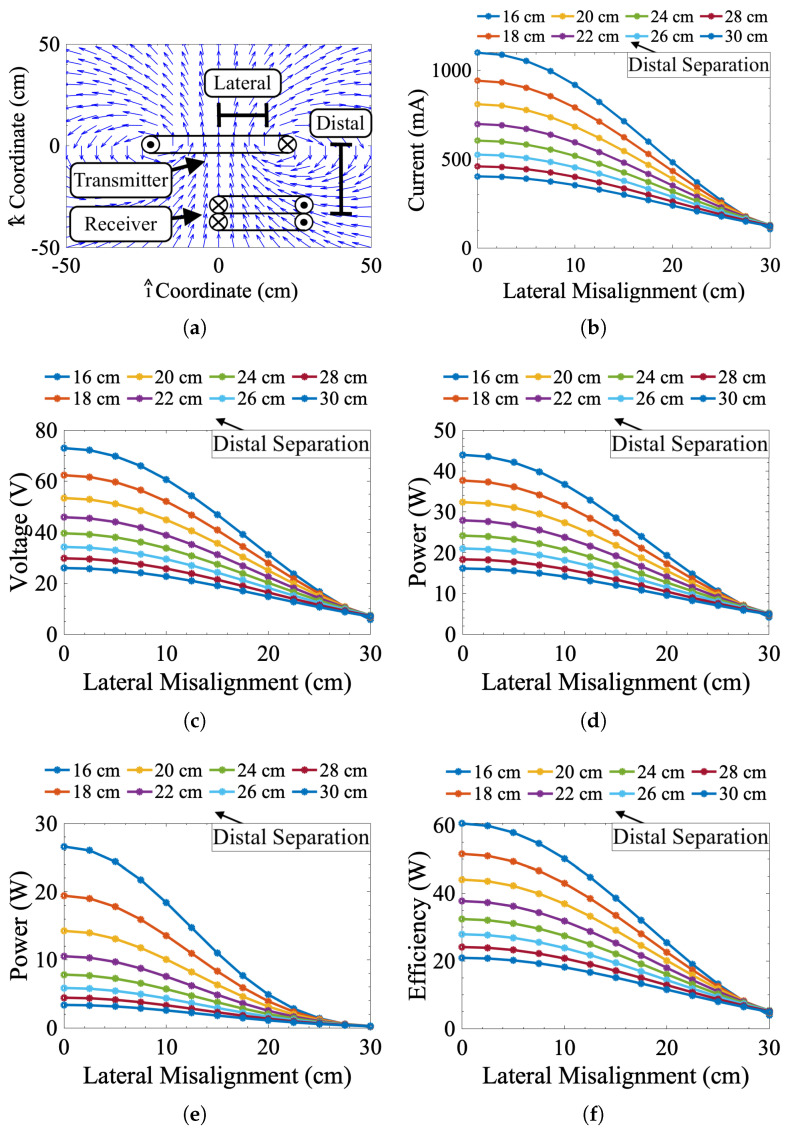
Simulated misalignment response data for 16 to 30 cm of distal separation and 0 to 30 cm of lateral misalignment. (**a**) Normalized vector plot of B(r) showing the location of the transmitter and receiver coils. (**b**) Transmitter current $I_{in}$, (**c**) receiver voltage $V_{Rout}$, (**d**) transmitted power $V_{in}\cdot I_{in}$, (**e**) received power $P_{out}$, and (**f**) efficiency η as functions of lateral (x-axis values) and distal (legend values) misalignment.

The efficiency η can be calculated using the following equation [32]:

(8) $$\eta =\frac{k^{2}\;Q_{p}\;Q_{s}}{{\left(1+\sqrt{1+k^{2}\;Q_{p}\;Q_{s})}\right)}^{2}}$$

where $Q_{p}$ and $Q_{s}$ are the quality factors of the primary and secondary coils, respectively. Figure 3 shows the numerically simulated misalignment response of this work’s IPT system using the nominal operating conditions in Table 1.

This model’s accuracy is discussed further in Section 3.2 of this paper, where the model’s output data are compared against measured values. However, there are some general trends that are expected in terms of prediction accuracy. For example, the Biot–Savart law used in Equation (1) is most accurate when applied to open spaces without nearby structures with high magnetic permeability. As such, the model treats the transmitter coil as a perfectly round closed loop in an open space. In reality, this coil is irregularly shaped and connects to a PCB with metallic heat sinks, RF connectors, and copper traces/pours. All of these factors are expected to distort the shape and magnitude of the magnetic field, as predicted by the Biot–Savart law, particularly when the coils are extremely close together, i.e., small distal separation. As distal separation increases, the model is expected to become more accurate since the receiver moves farther from the metallic structures, and the environment acts more like an open space. Based on this prediction, the model should be most accurate at 30 cm and least accurate at 16 cm of distal separation. Another factor to note with this model is the impact of complex media containing soil, water, and air. As mentioned earlier in this work, the relative permeability of these three materials is nearly identical and does not directly impact the shape and magnitude of the magnetic field predicted by the Biot–Savart law. However, wet soil is conductive, and the presence of a magnetic field can induce eddy currents in the soil. While this can produce some small secondary magnetic fields (Lenz’s law), the more prominent effect on the IPT transmission hardware will be increased loading on the transmitter, as reported in [33]. For the control signals, this effect will influence input current (lower η) but have little to no impact on receiver voltage.

### 2.2. Automated Alignment Approach

In multi-coil IPT architectures, additional coils provide extra information about the relative position of the primary and secondary power transfer coils. In particular, the sensing coils can provide information about the direction (i.e., $\hat{i}$, $\hat{j}$, and $\hat{j}$ contributions) of misalignment. With this information, an alignment robot could effectively align the coils using a standard feedback controller. Unfortunately, if the IPT system has a single coil pair, the measurement of current/voltage used to determine misalignment cannot be broken down into $\hat{i}$, $\hat{j}$, and $\hat{j}$ contributions, leading to a lack of state observability. By contrast, schemas drawn from the robot navigation and localization field seem better suited to this specific control problem. These navigation approaches are often used with ranging methodologies such as radio received signal strength indicators (RSSIs), which similarly struggle with issues of state observability [34]. Because transmitter current and receiver voltage do not give information about the direction of the misalignment, single-hypothesis approaches to localization, such as Kalman filtering, are not viable. Instead, a multi-hypothesis approach called a sequential importance sampling (SIS) particle filter was selected [35]. Fundamentally, this approach approximates the posterior probability distribution of some hidden Markov process’s internal states using weighted samples [36], i.e., “particles”. In this work, the internal state is just the location of the hidden coil. The following probability equation describes this particle filter:

(9) $$p\left(x_{k}|z_{1:k}\right)\approx \sum_{i=1}^{N_{s}}w_{k}^{i}\delta \left(x_{k}-x_{k}^{i}\right)$$

where $x_{k}$ represents the internal states of a Markov process that can be approximated using a series of observations, $z_{1:k}$ [36]. In this multi-hypothesis approach, $N_{s}$ samples, i.e., particles indexed by *i*, of $x_{k}^{i}$ represent estimates of $x_{k}$. Each hypothesis is assigned a significance weight $w_{k}^{i}$ for every discrete observation *k*. Particle weights are calculated using the following equation [36]:

(10) $$w_{k}^{i}\propto w_{k-1}^{i}\frac{p\left(z_{k}|x_{k}^{i}\right)p\left(x_{k}^{i}|x_{k-1}^{i}\right)}{q\left(x_{k}^{i}|x_{k-1}^{i},z_{k}\right)}$$

where $q\left(x_{k}^{i}|x_{k-1}^{i},z_{k}\right)$ is the importance density of the distribution, $p\left(x_{k}^{i}|x_{k-1}^{i}\right)$ is the transition prior probability of $x_{k}$, and $p\left(z_{k}|x_{k}^{i}\right)$ is the conditional probability of $z_{k}$ given $x_{k}^{i}$. Typically, this expression is reduced to:

(11) $$w_{k}^{i}\propto w_{k-1}^{i}\;p\left(z_{k}|x_{k}^{i}\right)$$

by selecting the prior probability distribution as the importance density [36]. By utilizing this machine-learning strategy, coil alignment can be achieved without needing to decompose measurements into directional contributions, i.e., eliminating the issue of unobservable states. Instead, alignment can be achieved by iteratively moving the mobile coil towards the most probable location of the hidden coil.

#### 2.2.1. Coil Alignment Particle Filter Formulation

In this work, coil alignment can be described as moving the position of a mobile coil $g_{k}$ to the particle location $x_{k}^{i}$ with the highest likelihood of containing a hidden coil $x_{k}^{i,\;best}$. The measurement $z_{k}$ is represented by the current and voltage measurements made during alignment. Particle weights are calculated by first finding the expected sensor measurements $z_{k,\;expected}^{i}$ at every particle using a model of z(r, h) such as the numerical model derived in this paper:

(12) $$z_{k,expected}^{i}=f\left(r_{k}^{i},\;h_{k}^{i}\right)$$

In this equation, $r_{k}^{i}$ and $h_{k}^{i}$ are the lateral and distal misalignments at each particle, respectively. For every particle, $r_{k}^{i}$ is the distance between $g_{k}$ and $x_{k}^{i}$:

(13) $$r_{k}^{i}=∥g_{k}-x_{k}^{i}∥$$

Once the expected sensor measurements are computed, the conditional probability of $z_{k}$ given $x_{k}^{i}$ can be found using the following equation:

(14) $$p\left(z_{k}|x_{k}^{i}\right)=\frac{1}{\sigma \sqrt{2\pi }}\exp\left[\frac{-1}{2\sigma^{2}}{\left(z_{k}-z_{k,expected}^{i}\right)}^{2}\right]$$

**Figure 4:**
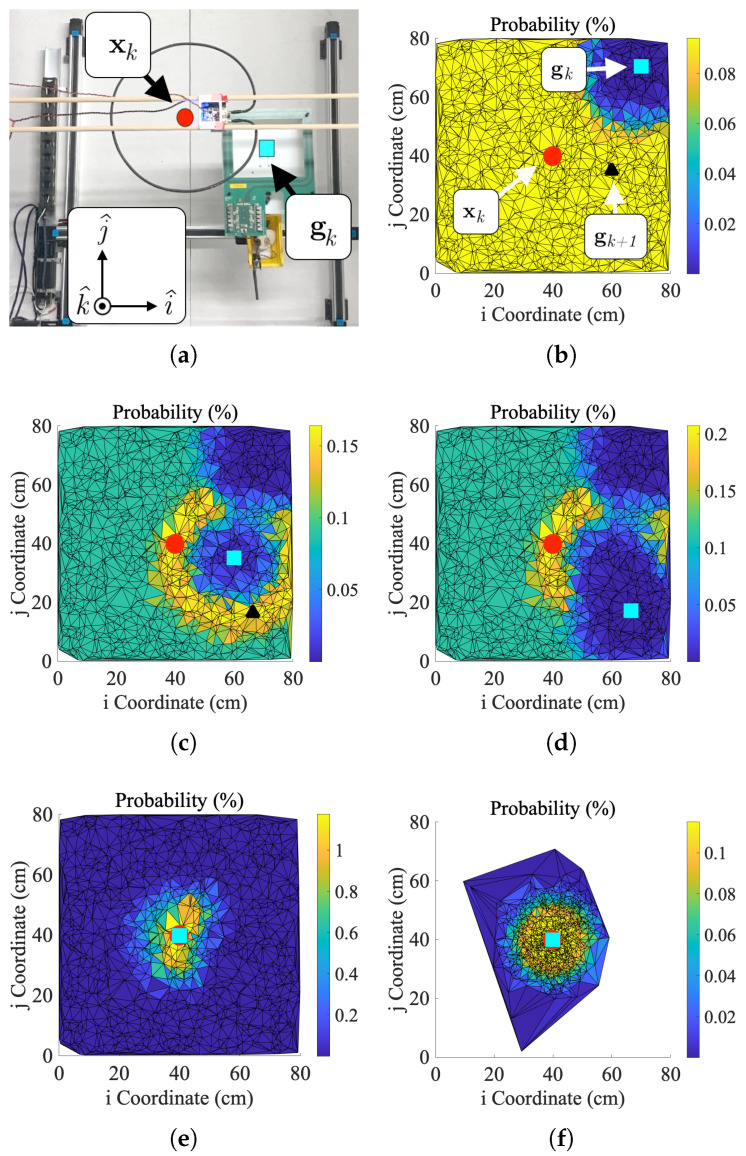
Particle filter tests. (**a**) Test setup with lateral misalignment in the $\hat{i}$ and $\hat{j}$ directions and distal misalignment in the $\hat{k}$ direction. (**b**) First, (**c**) second, (**d**) third, (**e**) fourth, and (**f**) fifth iterations of the alignment algorithm. The gantry’s current position ($g_{k}$), the best prediction of the hidden coil’s position ($g_{k+1}$), and the actual position of the hidden coil ($x_{k}$) are marked with cyan (square), black (triangle), and red (square) markers, respectively. The image in (**a**) corresponds to the second iteration of the particle filter. Iteration 1 is uncoupled. Resampling occurs after the fourth iteration.

In this computation, individual sensor measurements are assumed to be normally distributed due to noise with measurement variance $\sigma^{2}$. Figure 4 shows this process when applied to coil localization by depicting a mobile coil $g_{k}$ iteratively converging to the location of the hidden coil $x_{k}$.

#### 2.2.2. Particle Resampling

Resampling is a process in particle filtering in which new particles are drawn from an older set of particles. Low-weight particles from prior iterations are discarded to propagate higher-weight particles. The solution tends to be a better approximation due to the higher concentration of particles in high-likelihood positions. Particle filters incorporating resampling are often called sequential importance resampling (SIR) algorithms [36]. There are several standard resampling schemas, but a multinomial [37] approach was selected in this work due to ease of implementation. Resampling in the alignment particle filter produces a dense field of particles in areas where the hidden coil is most probably located. However, overuse of resampling prevents the particle filter from converging in an appreciable amount of time [36]. For this reason, the SIR particle filter in this work only resamples a single time when the sensor output exceeds some predetermined threshold value *Z*. The selection of *Z* can be somewhat arbitrary, but the effect of concentrating particles remains the same regardless. Resampling can be seen in Figure 4f. In this figure, the particles are resampled and concentrated around the center of the gantry’s workspace.

#### 2.2.3. The 3D Particle Filter Extension

In the previous particle filter formulations, $h_{k}^{i}$ has been held constant. However, knowing the exact distal separation between the IPT coils beforehand may be difficult or impossible in real applications of IPT. Instead of making an educated guess at some constant $h_{k}^{i}$, the SIS or SIR filter particles can be initialized using varying values of distal separation. Initializing particles in this manner allows for coil misalignment correction in all three spatial dimensions and helps correct minor inaccuracies in the parametric models of misalignment (modeling errors treated as an effective change in $h_{k}^{i}$). Unfortunately, maintaining the same particle spread as the two-dimensional approach would require $N_{s}^{3/2}$ particles (1000 particles per square meter in 2D or 31623 particles per cubic meter in 3D). This dramatic increase in particle volume is often called the “curse of dimensionality” in prior literature [36] since adding additional particle states frequently has this effect. Because each particle requires a minimum of 16 bytes of RAM ($\hat{i}$, $\hat{j}$, $\hat{k}$, and $w_{k-1}^{i}$, each stored as a 4-byte floating point number), large particle cloud volumes require tremendous amounts of memory. If memory is a limiting factor in particle initiation, the particles can instead be initialized on discrete planes in the $\hat{k}$ direction. While the number of planes used in the particle filter is somewhat arbitrary, with a good initial guess of distal coil separation, only a few planes should be necessary to achieve better alignment accuracy. Functionally, this approach is similar to the well-documented Fast-SLAM algorithm [38,39], where each particle tracks the probability of a known or discovered landmark. Here, each plane of distal separation is treated as an independent landmark, and the filtering algorithm successively decides which “landmark” is the most probable plane of distal separation. Accordingly, modeling errors or irregular coupling conditions will have the effect of weighing which “landmark” is most probable. So long as the altered misalignment response maintains the same general shape as anticipated by the empirical models, the 3D extension of the particle filter will always achieve the best alignment regardless of the altered conditions.

## 3. Results

### 3.1. Measured Misalignment Response

**Figure 5:**
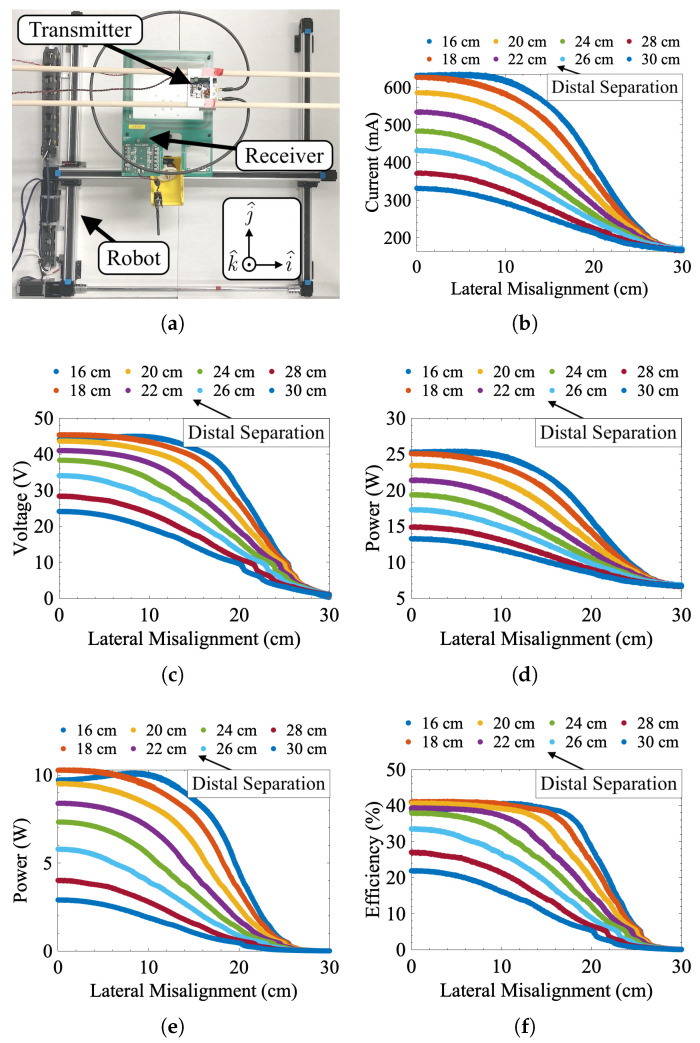
Measured misalignment response data. (**a**) Test setup with lateral ($\hat{i}$ and $\hat{j}$ plane) and distal ($\hat{k}$ position) misalignment. (**b**) Transmitter current, (**c**) receiver voltage, (**d**) transmitted power, (**e**) received power, and (**f**) transmission efficiency as functions of lateral (x-axis values) and distal (legend values) misalignment.

The misalignment characterization tests involved a set of eight linear sweeps to gather IPT misalignment data for different values of lateral and distal misalignment. These tests were conducted to validate the accuracy of the numerical model and provide a means of generating an empirical model if the numerical approach proved too inaccurate. To facilitate this task, a large (80 by 80 cm workspace), custom-designed Cartesian robot (see Figure 5a) was built to precisely control the alignment of two IPT coils while taking sensor measurements. Parametric sweeps were run on this platform by first mounting the 13.56 MHz IPT system’s receiver on the Cartesian robot. The coupled IPT transmitter was fixed some adjustable distance away from the receiver. Each sweep consisted of exciting the transmitter at a fixed voltage of 40 VDC and moving the receiver to simulate lateral misalignment. While the receiver moved, corresponding changes in input current and rectified voltage were measured. Each 1 mm resolution sweep advanced such that the Cartesian robot moved in a straight line directly over the location of peak alignment between the IPT coils. The location of peak alignment was found using a high-precision grid sweep and interpolation. These linear sweeps gathered data at 16, 18, 20, 22, 24, 26, 28, and 30 cm distal separation between the transmitter and receiver coils. Data were gathered for the linear sweeps by running each sweep multiple times and averaging them together for every value of distal separation. Figure 5b–f shows the results of these sweeps. Overall, reducing misalignment increased the induced voltage estimate, power transmission magnitude, and efficiency. At 16 cm of distal separation, the IPT system acts anomalously where efficiency and received power actually decrease at peak concentric alignment. This effect is not unexpected and is likely due to the previously mentioned effects of nonideal environments, particularly magnetic coupling with the metallic structures of the alignment robot.

### 3.2. Numerically Modeled vs. Measured Data

**Figure 6:**
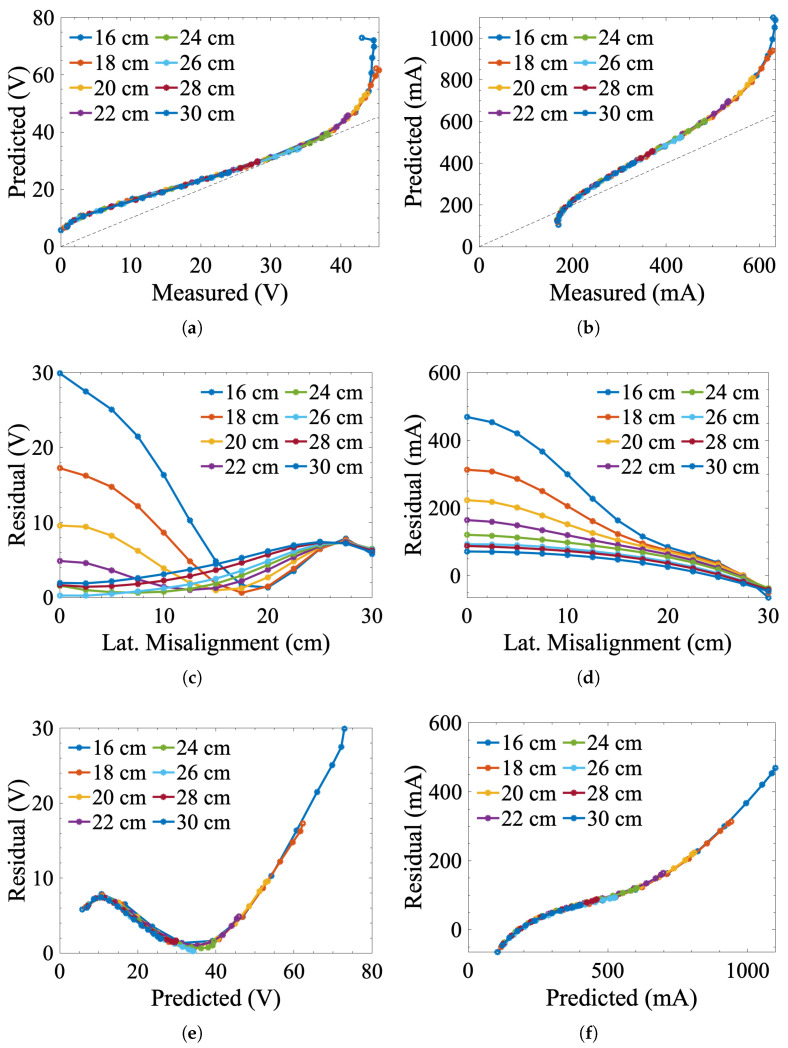
Error analysis of the theoretical model: (**a**) receiver voltage and (**b**) transmitter current measured vs. predicted plots for different values of distal separation. Note the gray line in these figures represents the line y=x+0, i.e., the expected trendline for perfect correlation between measured and expected values. Subfigures (**c**–**f**) show the residual errors of $z_{predicted}-z_{observed}$ for both control signals as functions of lateral misalignment and predicted output signals.

The measured control signal results were compared against data from the numerical model discussed in Section 2 by analyzing residual errors. Ideally, the numerical data should map directly to the measured results, i.e., $z_{predicted}-z_{observed}=0$. Figure 6 shows “measured-vs.-predicted” plots for each value of distal separation and the corresponding residual plots as functions of the predicted control signals and lateral misalignment. Table 2 shows the root mean square error (RMSE) of the model for different values of distal separation.

For the voltage control signals, errors are relatively high at low values of distal separation (16, 18, and 20 cm) but relatively low at greater coil separations (22, 24, 26, and 30 cm). Overall, the model tends to overpredict the voltage signals, particularly when the IPT coils are extremely close together. The current signals show the same general trends as the voltage signals. These trends are not unexpected and are likely due to magnetic field distortions mentioned earlier in this paper. The greater errors with the current-based control signals are also not unexpected, given that Equation (8) does not consider factors such as switching losses that increase inefficiency. Based on the results in Figure 5, efficiency was observed to change with both distal and lateral misalignment. Interestingly, prediction errors tend to also increase at high values of lateral misalignment, i.e., where control signal magnitude should be the smallest. This effect is most likely observed due to forward power losses in the rectifier diodes. At lower voltages, the diodes used in the receiver’s rectifier will significantly affect the voltage signal due to their forward voltage drops. Regardless of these issues, the numerical model effectively predicts the control signals’ overall general behaviors. Reducing lateral and distal misalignment for both signals improves coupling and results in larger control signal magnitudes. For all but the closest values of distal separation, the predicted control signals only vary by a few volts or 10 s of milliamps. Furthermore, the model accurately predicts the IPT system’s maximal tolerance to lateral misalignment (30 cm).

### 3.3. Emperical Modeling

Despite the capabilities of the numerical model, there are several key disadvantages to using this approach in an alignment control system. While the statistical-based alignment approach should be quite tolerant of minor modeling errors, the errors observed from the numerical model are likely too large for effective implementation in the alignment algorithm. For this reason, empirical models of misalignment generated using parametric sweeps may be better suited to the problem of alignment correction in field settings due to their inherent capture of behaviors caused by irregular environmental conditions. Empirical models of the misalignment were created by fitting the linear sweep data to a Fourier fit described by the following equation:

(15) $$z\left(r\right)=a_{0}+a_{1}\cos\left(\omega r+b_{1}\right)+a_{2}\cos\left(2\omega r+b_{2}\right)$$

In the above equation, $z\left(r\right)$, the transmitter current or receiver voltage, is represented as a function of lateral misalignment *r*, a fit frequency ω, and constants *a* and *b*. Data were fitted to this equation over the range of 0 to 30 cm of lateral misalignment. The resulting equations were coupled with an interpolation algorithm to predict $z\left(r,h\right)$ for values of distal separation *h* that were never explicitly measured in testing. These empirical models have the added advantage of accounting for any and all potential sources of modeling error, e.g., irregular coil shapes, heating, and parasitic coupling with ferromagnetic structures in a given environment. Such adaptive capabilities are a necessity in field settings or when applied to complex structures due to their often unpredictable effect on the distribution of the magnetic field about the transmit coil. A Fourier fit was selected due to the form of analytic solutions published in prior literature concerning IPT coil misalignment [40,41]. While those closed-form solutions tend to have a narrower range of applicability, many works have found success in modeling IPT coil misalignment trigonometric functions. Additionally, through trial and error, even simple two-frequency Fourier fits provided more accuracy than high-order polynomial fits. Based on the RMSE values shown in Table 3, the empirical model demonstrated an average RMSE of only 1.77 mA and 0.255 V for the control signals relative to what was measured during the parametric sweeps. By contrast, the numerical models had an average RMSE of 118 mA and 6.74 V. When directly compared, the empirical model is roughly 67× and 26× more accurate than the numerical model for current and voltage-based control signals, respectively.

### 3.4. Source of Numerical Modeling Error

**Figure 7:**
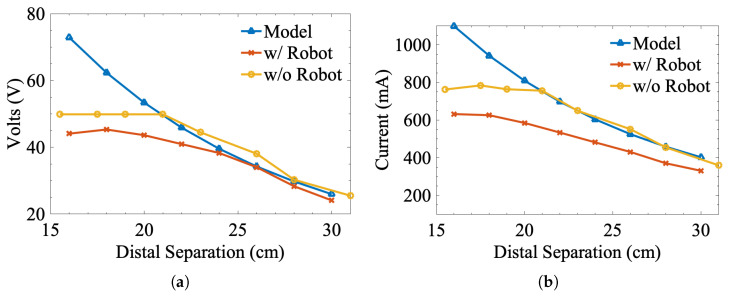
Control signal comparison. Each subfigure shows the (**a**) voltage and (**b**) current-based control signals based on predictions from the numerical model, measurements made with the robot present, and measurements made without the robot present.

While the empirical models developed in this work are a much more accurate representation of the misalignment response, understanding the sources of error in the numerical model could lead to the development of more accurate simulations. As mentioned throughout this paper, the initial suspicion was that parasitic coupling with nearby metallic structures was driving up the error with our numerical model. While isolating the coils from all nearby metallic structures was not feasible, e.g., pipes under the floor and nearby tables, we conducted power transmission tests with and without the robot present. In these tests, the coils were held at peak concentric lateral alignment and energized. The distal separation between the coils was increased after each iteration of testing. In tests without the robot, the elevation of the receiver coil was adjusted using high-density polyethylene spacers. Figure 7 shows the results of these tests by plotting the control signals predicted using the empirical models against the signals measured with and without the robot. From the data in these plots, the presence of the robot clearly has a measurable effect on the misalignment response and corresponding control signals. While the response without the robot does not match the numerical model perfectly, the differences measured during these tests were significantly lower than the differences when the robot was present. Interestingly, there is a plateau effect on the control signals at low values of distal separation. This may be due to nonideal behavior on the Class-EF inverter, though verifying this requires further investigation. However, overall, parasitic coupling with ferromagnetic structures is a significant source of error in our numerical model. Unfortunately, this effect is difficult to account for using the numerical modeling approach discussed in this work, given the highly irregular shape and size of the Cartesian robot and the agricultural vehicle used later. As such, empirical modeling is a necessity in complex environments with metallic structures.

### 3.5. Benchmarking Alignment Algorithm Performance

**Figure 8:**
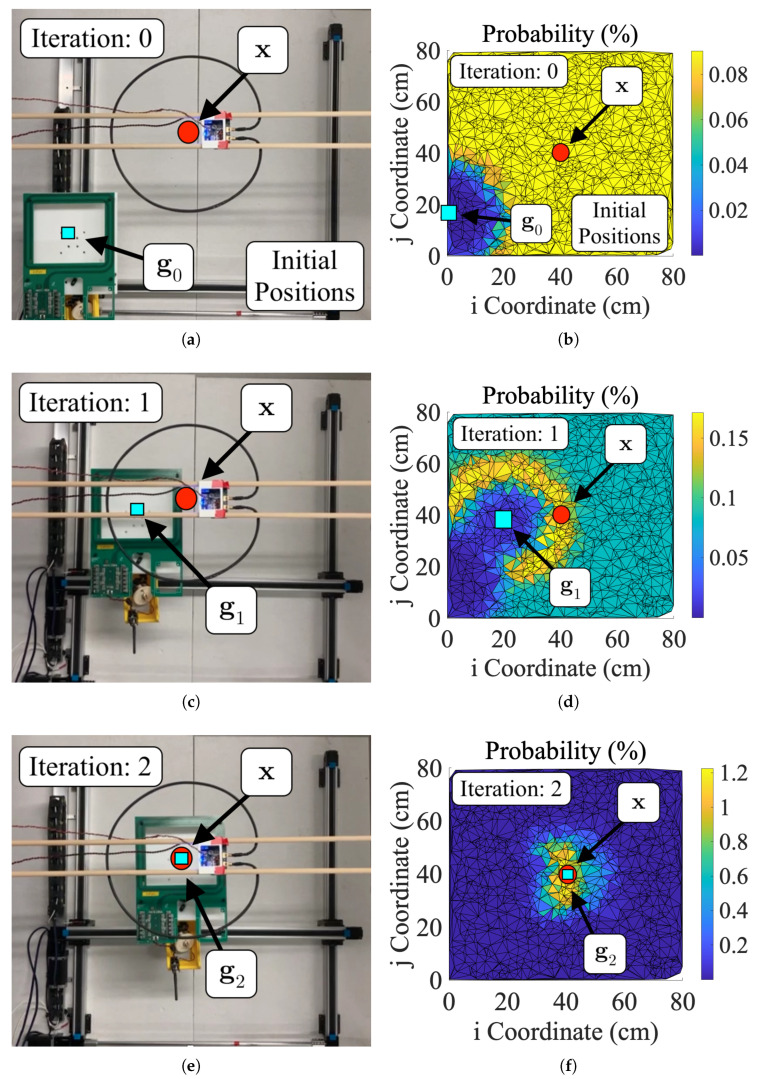
Sample data and images from a coil alignment test. Subfigures (**a**,**b**) show the test setup and probability map for an initially uncoupled test. Collectively, these figures represent iteration “zero” of the particle filter as the robot has not actually moved but has updated probabilities once. Subfigures (**c**,**d**) show data from iteration “one” of the test cycle. Similarly, subfigures (**e**,**f**) show data from iteration “two” of the test cycle, i.e., the final iteration. While initially uncoupled tests usually take more than three measurements to converge, this particular test cycle was simply lucky in its predictions of $g_{k+1}$.

To characterize the performance of the IPT system with particle filter misalignment correction, the test setup shown in Figure 5 was programmed to run the particle filters described in Section 2. The receiver coil position $g_{k}$ was initialized randomly in the Cartesian robot’s 2D workspace for all alignment tests. Distal separation was fixed at 19.75 cm. Due to the randomness of initialization, some IPT tests were initially coupled, and others were uncoupled, i.e., greater than 30 cm lateral coil misalignment. Each particle filter was programmed with a fixed particle count $N_{s}$ of 1875. Once initialized, the Cartesian robot used various particle filters to align the receiver with the fixed, stationary transmitter. The filters computed probabilities (selection of $z_{k}$ variables) using transmitter current, receiver voltage, or a combination of both and the empirical models of the control signals. The combination approach computes $w_{k}^{i}$ for current and voltage. These independent weights were then multiplied together and normalized. The entire process can be visualized using Figure 8. Although in these tests, the receiver is technically the mobile half of the IPT system, the process is fundamentally identical to using a mobile transmitter so long as the current-based control signal is used for computing probabilities. Table 4 summarizes filter performance from these tests. The values shown in this table represent the achieved power transmission performance at peak achieved alignment. Table 5 compares the results of tests using different control signals (voltage, current, or combined) and particle filter types (SIR, SIS, 2D, and 3D). Each comparison is made using two-sample *t*-tests.

## 4. Discussion

### 4.1. Mobile Transmitter vs. Mobile Receiver Localization Capabilities

Based on the data in Table 5, the SIS particle filters did not differ significantly in achieved transmission efficiency or speed when using current (mobile transmitter perception method) or voltage (mobile receiver perception method) to perceive misalignments. The current-based SIR filter was statistically worse than the voltage-based SIR filter in terms of speed and efficiency. These discrepancies are likely due to how the resampling threshold *Z* efficiency was selected. In both sets of tests, *Z* was fixed at a certain percentage of the sensor’s full-scale range (2%). In retrospect, these values map to slightly different lateral misalignments, meaning resampling occurs at other times in the alignment process for the different algorithms. However, despite any differences, the effect size is not large (less than 1% efficiency and 1 iteration), meaning that any statistically derived differences are negligible in practice. Consequently, the particle filters should work using either a mobile transmitter or receiver, i.e., transmitter current or induced voltage as $z_{k}$, without any notable change in performance.

The combined method of computing $P(x_{k}|z_{k})$ was less efficient than the current or voltage-based filters. This result is almost certainly a consequence of accelerated sample degeneracy. The current and voltage measurements are related to the same metric of induced voltage. When combining their probabilities, certainty increases without novel information, causing degeneracy. Still, despite differences in performance, the effect size is still not large, and any decrease in performance is not practically meaningful. This outcome demonstrates that fused sensor data are not necessarily better than the independent misalignment estimates for this particle filter formulation.

### 4.2. Performance Metrics: Accuracy and Speed

Alignment accuracy can be represented in terms of either geometry (overlap of the coils’ centroids) or performance (expected vs. achieved performance). Realistically, geometric accuracy is difficult to measure during testing and may or may not correspond to optimal performance conditions, especially in field settings. Instead, this work uses a performance-based metric by comparing expected (from the linear sweep-based empirical model) and achieved power transfer efficiency. Once computed in terms of efficiency, alignment accuracy in terms of distance can be backtracked using the empirical models formulated in this work. Similarly, speed can be defined in two different ways: iterative (iterations until peak achieved alignment) or literal (time until peak achieved alignment) speed. The iterative metric is a better performance measure because it is invariant of the alignment robot’s speed capabilities. Accuracy and speed performance metrics are summarized in Table 4.

For a distal separation of 19.75 cm, the parametric model predicts a maximum achievable efficiency of 40.8%. Averaging the outcomes of all six filter types (see Table 4, excluding data from the combined probability approach) results in a nominally worse efficiency value of 40.7%. This slight reduction in efficiency corresponds to just under 1 cm misalignment, i.e., 0.025 coil diameters, at 19.75 cm of distal separation. Interestingly, the best-performing particle filters achieved power transmission efficiencies slightly greater than the model prediction, likely due to minor model inaccuracies.

Using the iterative metric and the same six filter types, on average, the filters took 3.45 iterations to localize the hidden coil when initially coupled and 3.92 iterations regardless of coupling. Literal alignment speed was, on average, 11.7 s regardless of coupling. Given an average initial misalignment of 31.5 cm, an effective speed *v* can be defined as:

(16) $$v=\frac{|g_{\mathrm{Final}}-g_{\mathrm{Start}}|}{{\mathrm{Time}}_{\mathrm{Final}}-{\mathrm{Time}}_{\mathrm{Start}}}$$

and was benchmarked at 2.69 cm/s. Unlike iterative speed, literal speed is a function of the alignment robot and can be altered significantly with software modifications of the robot (programmed maximum speed of 3.00 cm/s during testing).

### 4.3. Field Testing

**Figure 9:**
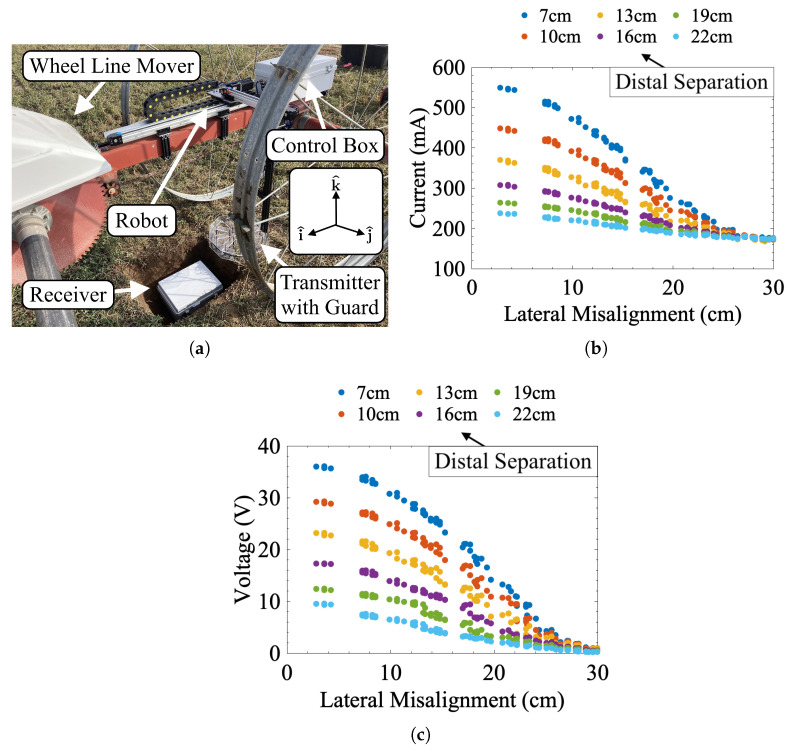
Summary of field testing: (**a**) test setup, (**b**) transmitter current, and (**c**) receiver voltage as functions of lateral (x-axis values) and distal (legend values) misalignment. Note that the values of distal misalignment denote the distance between the transmitter and the top layer of soil. For the coil-to-coil distance add an additional 13 cm (10 cm soil and 3 cm air inside the receiver).

After laboratory testing, the hardware used in this work was integrated into an agricultural soil sensing platform to demonstrate the effectiveness of this misalignment perception and correction routine in real-world applications. The building blocks of this platform have been previously discussed in [33]. Fundamentally, this platform consists of a buried IPT receiver attached to multiple soil sensors. An aboveground power transmission system recharges the underground hardware and collects sensor data. Prior works faced two related issues during testing that remained largely unresolved: complete vehicle integration and misalignment correction [33]. For these field tests, the IPT system and misalignment correction hardware were mounted on a wheel line (see Figure 9a) at the Utah Agricultural Experiment Station in Kaysville, Utah. Wheel lines are pieces of agricultural equipment used in conjunction with water wells for surface irrigating crops. Operationally, a wheel line is typically moved over a planted field, and sprinklers attached to the line spray water on nearby crops. Modern wheel lines are generally moved through a field using a gas or solar-powered vehicle referred to as a wheel line mover. Most movers are only capable of 1D forward and reverse motion, though the wheels of the mover and line often form tracks in the soil that prevent lateral motion. Based on the coordinate space established in Figure 9a, the wheel line is capable of only coarse motion in the $\hat{i}$ direction, while the correction hardware can move finely in the $\hat{i}$ or $\hat{j}$, i.e., the same degrees of freedom as the benchtop setup. The accompanying IPT receiver box was buried under 10 cm of soil for these initial tests, though greater depths are likely possible. Once the receiver was underground, a series of sweeps similar to those in Section 2 were conducted to generate a parametric model of misalignment regarding transmitter current, receiver voltage, and lateral/distal misalignment (see Figure 9b,c for the response data). In these sweeps, the wheel line mover was parked in the general area of the underground installation. The supercapacitors and sensors were left disconnected from the receiver to reduce the number of confounding variables during alignment testing. Once parked, the aboveground alignment hardware was powered using a battery bank and driven in a grid sweep pattern to gather parametric data. These data were then aggregated and fitted into a Fourier fit model using Equation (15).

Once a parametric model of misalignment was constructed, a series of ten SIS filter particle tests were run to align the aboveground transmitter with the obstructed underground receiver. On average, the automated alignment algorithm converged after 4.00 iterations on average, which is slightly slower than the results reported during laboratory testing. While convergence speed is slower, the difference between 3.92 iterations and 4.00 is negligible in this application. Furthermore, even moving the Cartesian robot to a single additional position does not take significant time or other resources, i.e., power or fuel. On average, the IPT system and correction hardware could transmit 7.52 W of power at 32.0% efficiency at peak concentric alignment and 19.75 cm of distal separation. At this rate, 2.4 min of recharging (1083 J) could theoretically power existing [42] soil monitoring technology for over a year (dormant, no measurements) or empower that same technology to take 13,000 measurements. Interestingly, the efficiency values achieved during field testing are actually 1.5% higher than expected from the empirical models, likely due to varying environmental conditions throughout the day. As such, the error using the metric defined in this work was fundamentally 0 cm. This outcome demonstrates that the alignment algorithm can be implemented in field settings without a meaningful change in performance.

### 4.4. Adaptations for Select IPT Topologies

The work presented throughout this paper focused on building the perception and misalignment correction methodologies around a Class EF-driven IPT system. Despite this emphasis, the underlying method of mapping misalignments to changes in inductive coupling should be effective for almost any inductive power transfer system, with some slight adjustments to the control signals used for coil positioning. Interestingly, with many IPT topologies, little to no adaptation would be required to implement the perception and correction strategies proposed in this work. Because induced voltage fundamentally maps to inductive coupling, this signal can be used for misalignment mapping on virtually any IPT system. Similarly, for most resonant inverter-fed topologies [43], such as Class E [44], D/DE [45], and EF_*n*_ [16], the control signal of input current maps to inductive coupling/misalignment as well. While these are certainly not the only control signals that map to coupling/misalignment on IPT hardware, the ones discussed in this work directly apply to a wide range of IPT systems.

## 5. Conclusions

This paper presents a novel method of sensing and correcting coil misalignment for a Class EF-driven inductive power transfer system for recharging underground sensor networks. The empirical model of the misalignment response proved to be both accurate and capable of rapid reformulation to accommodate diverse environmental conditions that impact inductive coupling. The alignment methodology corrects, rather than compensates for, misalignment and eliminates the need for complex multi-coil measurement systems. Furthermore, this approach is equally capable of aligning using either half (transmitter or receiver) of the IPT system independently and without data telemetry. Based on these results, this alignment methodology could theoretically be used with a mobile transmitter or receiver with equal effectiveness. Field testing in this work successfully demonstrated the applicability of this perception and correction methodology in real-world sensor applications. Although only tested on a Class EF-driven IPT system, the underlying misalignment sensing approach should work for various common IPT architectures, including Class E and Class D-driven systems, with minimal adaptation to the transmitter and receiver setups. Overall, this work contributes to the field of inaccessible sensor networks by providing a robust recharging solution that features automated correction of the power delivery hardware.

## Figures and Tables

**Table 1:** Operating parameters for the 13.56 MHz, Class EF-driven IPT system.

Parameter	Symbol	Value
Turns in Receiver Coil	*N*	2
Transmission Frequency	ω, f	85.2 Mrad/s, 13.56 MHz
Current in Primary Coil	$i_{p,rms}$	6.25 A
Primary Coil Self-Inductance	$L_{p}$	1181 nF
Secondary Coil Self-Inductance	$L_{s}$	2470 nF
DC Input Voltage	$V_{in}$	40 V
Output Load	$R_{out}$	200 Ω
Quality Factor	$Q_{p}$, $Q_{s}$	767

**Table 2:** Root mean square error (RMSE) of the numerical model’s signals benchmarked against measured signals.

Distal Separation (cm)	RMSE Voltage Signal (V)	RMSE Current Signal (mA)
16	15.9	268
18	9.55	185
20	6.09	135
22	4.48	104
24	4.08	82.2
26	4.22	65.6
28	4.66	60.5
30	4.96	50.6

**Table 3:** Root mean square error (RMSE) of the empirical model’s signals benchmarked against measured signals.

Distal Separation (cm)	RMSE Voltage Signal (V)	RMSE Current Signal (mA)
16	0.397	3.19
18	0.256	2.64
20	0.245	2.25
22	0.224	1.35
24	0.224	1.63
26	0.216	0.938
28	0.235	1.17
30	0.240	0.986

**Table 4:** Alignment algorithm performance metrics in terms of alignment error, efficiency η, and iterative speed. This table includes the frequency of initially uncoupled (IU) and coupled (IC) tests. Results are shown for current (*I*), voltage (*V*), and combined (*C*) methods of calculating probabilities. Note the expected efficiency at peak concentric alignment, and 19.75 cm distal separation is 40.8%, meaning some tests exceeded the expected accuracy, likely due to minor modeling errors. Such tests are specially marked (*). Also, note that speed values are only reported for initially coupled tests due to the randomness in initially uncoupled tests.

Filter	IU, IC	Avg. Error	Avg. η	Std. η	Avg. Speed	Std. Speed
2D SIS, *I*	59, 41	3.8 cm	40.3%	0.974%	3.10	1.05
2D SIS, *V*	56, 44	2.4 cm	40.5%	0.819%	2.86	1.10
2D SIS, *C*	57, 43	8.3 cm	39.9%	0.952%	2.79	1.67
2D SIR, *I*	26, 24	3.1 cm	40.4%	1.04%	4.00	1.47
*2D SIR, *V*	34, 16	0.0 cm	41.2%	0.966%	3.13	1.15
*3D SIS, *I*	20, 30	0.0 cm	41.0%	1.16%	3.80	1.65
*3D SIS, *V*	35, 15	0.0 cm	41.0%	1.06%	3.80	2.48

**Table 5:** Statistical evaluation of current (*I*) vs. voltage (*V*) vs. combined (*C*) methods of misalignment perception. Mean differences (difference), degrees of freedom (df), and *p*-values are shown for each *t*-test.

Compare	Filter	Metric	Difference	df	*p*-Value
*I* vs. *V*	2D SIS	η	−0.186%	192	.146
		Speed	0.234	60	.500
	2D SIR	η	−0.846%	98	<.001
		Speed	0.875	37	.0422
	3D SIS	η	0.0651%	97	.771
		Speed	0.00	20	1.00
*I* vs. *C*	2D SIS	η	0.443%	198	.00132
		Speed	0.307	79	.443
*V* vs. *C*	2D SIS	η	0.629%	194	<.001
		Speed	0.0729	70	.808

## Data Availability

The dataset used during this study can be obtained by contacting the corresponding authors and agreeing to terms and conditions.

## Abbreviations

The following abbreviations are used in this manuscript:

WSUN	Wireless underground sensor network
IPT	Inductive power transfer
SIS	Sequential importance sampling
SIR	Sequential Importance resampling
WPT	Wireless power transfer
SLAM	Simultaneous localization and mapping
